# Potential activities and mechanisms of extracellular polysaccharopeptides from fermented *Trametes versicolor* on regulating glucose homeostasis in insulin-resistant HepG2 cells

**DOI:** 10.1371/journal.pone.0201131

**Published:** 2018-07-19

**Authors:** Ju-Fang Teng, Chien-Hsing Lee, Tai-Hao Hsu, Hui-Chen Lo

**Affiliations:** 1 Department of Nutritional Science, Fu Jen Catholic University, New Taipei City, Taiwan; 2 School of Chinese Medicine, College of Chinese Medicine, China Medical University, Taichung, Taiwan; 3 Division of Pediatric Surgery, Department of Surgery, China Medical University Children’s Hospital, Taichung, Taiwan; 4 Department of Bioindustry Technology and Department of Medicinal Botanicals and Health Care, Da-Yeh University, Changhua, Taiwan; Stellenbosch University, SOUTH AFRICA

## Abstract

Polysaccharides derived from mushrooms have potential to control blood sugar, reduce insulin resistance and prevent diabetic complications. The intracellular polysaccharopeptides of *Trametes versicolor* (TV) have been used as immunologic and oncologic adjuvants. The aim of this study was to investigate the potential activities and mechanisms of extracellular polysaccharopeptides (ePSP) obtained from TV strain LH-1 on regulating glucose homeostasis. Human hepatoma HepG2 cells incubated with normal glucose (5.5 mM, NG model), high glucose (33 mM, HG model), or high glucose (33 mM) plus high insulin (10^−7^ M, HGI model) concentrations were administered with TV LH-1 ePSP (50, 100, and 1000 μg/ml) for 24 hr. Glucose uptake of HepG2 cells, determined by flow cytometry, was significantly decreased in the HG and HGI models with insulin stimulation, suggesting insulin resistance of these cells; however, ePSP reversed this decrease in a dose-dependent manner (one-way ANOVA, p<0.05). In the HG and HGI models, ePSP significantly increased glycogen content, insulin receptor substrate-2 protein and phosphorylated AMP-activated protein kinase (AMPK), as determined by western blot analysis. In addition, ePSP significantly increased glucokinase in the NG and HG models, increased membrane glucose transporter-1 and decreased glycogen synthase kinase-3β in the HGI model, and increased glucose-6-phosphatase in the NG and HGI models (one-way ANOVA, p<0.05). In summary, TV LH-1 ePSP may elevate cellular glucose uptake to regulate glucose homeostasis via the activation of AMPK and glycogen synthesis in an insulin-independent manner. These results suggest that TV LH-1 ePSP may be a nutraceutical with anti-hyperglycemic activity.

## Introduction

Diabetes mellitus (DM) is a chronic metabolic disease that has become a substantial economic burden worldwide. It has been reported that more than 10% of health-care dollars are directly attributable to diabetes in USA [1]. In Taiwan, patients with diabetes had 1.4-fold higher medical costs from the national health-care system than patients without diabetes [2]. The prevalence of DM is persistently increasing worldwide, which increases the global medical costs. The use of dietary supplements, nutraceuticals, and functional foods is considered as an alternative approach to prevent hyperglycemia and attenuate diabetes-associated complications [3]. Several mushrooms have been demonstrated to have anti-hyperglycemic and anti-diabetic activities for their components such as polysaccharides and other constituents [4]. However, the mechanism of polysaccharides in modulating glycemic control is still under investigation.

*Trametes versicolor* (L.:Fr.) Pilát (TV), formerly known as *Coriolus versicolor*, has been found to have various biological activities, including hepatoprotective [5], anti-inflammatory [6], antioxidant [7], anti-genotoxic [8], immunoregulatory [9], anti-tumor [10], and anti-viral [11] abilities. The best known commercial preparations of TV are intracellular polysaccharopeptides, such as Krestin (PSK) and polysaccharopeptide (PSP). PSK and PSP have been used as chemoimmunotherapy agents in the treatment of cancer, which are derived from the CM-101 and COV-1 strains of TV by Japanese and Chinese, respectively [10]. A novel TV strain LH-1, originally collected in Taiwan, has been successfully cultured in submerged fermentation to produce copious amounts of extracellular polysaccharopeptides (ePSP). TV LH-1 ePSP has been proven to be safe for oral consumption in an animal study [12]. In addition, the immunomodulatory [13] and bone protective properties [14] of TV LH-1 ePSP have been demonstrated in cell and animal studies. It is reasonable to believe that TV LH-1 ePSP may have great potential in regulating glucose homeostasis for its high relative percentages of D-glucose and D-galactose in polysaccharides that is similar to the intracellular polysaccharides found with alpha-glucosidase inhibitory properties [15].

For screening anti-hyperglycemic agents, *in vitro* models using hepatocytes, the key cells in regulating glucose metabolism, are crucial in dealing with large numbers of preliminary products. Human hepatoma HepG2 cells that maintain liver cell morphology and function have been cultured with high glucose, with or without high insulin levels, to mimic the different diabetic conditions, such as type 1 and type 2 DM [16]. To determine glucose uptake of live cells, several studies used fluorescent labeled, non-radioactive D-glucose analog 2-(N-(7-nitrobenz-2-oxa-1,3-diazol-4-yl)amino)-2-deoxyglucose (2-NBDG) as an alternative to radio-labeled D-glucose and quantified the percentage of cells with fluorescence by flow cytometer [17, 18]. 2-NBDG is a non-isotopic, relatively safe and sensitive *in vitro* tool which can be incorporated into living cells via the glucose transporting system to assess glucose uptak. Using HepG2 cells and 2-NBDG, we aimed to evaluate the potential activities and mechanisms of TV LH-1 ePSP on regulating glucose homeostasis by determining the glucose uptake and proteins involved in the insulin signaling pathway, glucose transporters, glycolysis, gluconeogenesis, glycogenesis, and energy homeostasis, i.e., AMP-activated protein kinase (AMPK) activation [19].

## Materials and methods

### Preparation of extracellular polysaccharopeptides (ePSP) from fermentation cultures of TV LH-1

TV LH-1 collected from Nantou, Taiwan was maintained on potato dextrose agar plates and seeded and fermented in culture medium (4.0% glucose, 0.3% peptone, 0.15% KH_2_PO_4_, and 0.15% MgSO_4_·7H_2_O) at 25°C, pH 4.5–5.0, 100 rpm for 7 days in 20 liters of fermenter (Bio-top, Taiwan) as described in previous studies [13, 20]. The mycelium was removed by centrifugation, and the supernatant was precipitated with 95% ethanol at 4°C overnight and centrifuged at 11,000 ×g for 20 min to obtain TV LH-1 ePSP. The contents of total sugars and proteins and the monosaccharide composition of TV LH-1 ePSP were reported in the previous study [13, 15]. In brief, each gram of TV LH-1 ePSP contained 138 mg of protein, 82.27 mg of glucose, 8.67 mg of galactose, 8.18 mg of mannose and 0.87 mg of xylose [13].

### Cell culture

HepG2 cell line (BCRC 60025) purchased from the Food Industry Research and Development Institute of Taiwan were cultured in Dulbecco’s Modified Eagle Medium (DMEM, GIBCO, CA, USA) with 10% fetal bovine serum (FBS) in a humidified atmosphere of 5% CO_2_ at 37°C. Cells were seeded into 6-well (5×10^5^ cells/well), 24-well (1×10^5^ cells/well) and 96-well (1×10^4^ cells/well) plates and 100-mm dishes (4×10^6^ cells/dish) for glycogen amount, glucose uptake, cell viability, and protein extraction, respectively.

### The establishment of normal glucose (NG), high glucose (HG), and high glucose plus high insulin (HGI) models

HepG2 cells were seeded in 24-well plates with DMEM culture medium containing 5.5 mM glucose and 10% FBS and incubated in a humidified atmosphere of 5% CO2 at 37°C. After 24 hr of incubation, the medium was removed and replaced by that containing 1% FBS and 5.5 mM glucose for the normal glucose (NG) model, 33 mM glucose medium for the high glucose (HG) model and 33 mM glucose plus 10^−7^ M insulin for the high glucose, high insulin (HGI) model [21, 22].

### Cell viability

HepG2 cells were seeded in 96-well plates with DMEM culture medium containing 5.5 mM glucose and 10% FBS and incubated in a humidified atmosphere of 5% CO2 at 37°C. After incubation for 24 hr, the medium was removed and replaced by those containing 5.5 mM glucose, 10% FBS and 0, 0.01, 0.1, 1, 10, 100, 500, 1000, 2000, or 4000 μg/ml of PSK or TV LH-1 ePSP. A commercial product of TV intracellular polysaccharopeptides, PSK is consumed as a functional food to prevent and alleviate hyperglycemia in China and Taiwan and, in the present study, used as a control to assess the activity of TV LH-1 ePSP on regulating glucose homeostasis. After incubation for 24, 48, and 72 h, cell viability was determined using 3-(4,5-dimethylthiazol-2-yl)-5-(3-carboxymethoxyphenyl)-2-(4-sulfophenyl)-2H-tetrazolium inner salt (MTS) commercial kit (CellTiter 96® AQueous One Solution Reagent, Promega, WI, USA) according to the assay protocol.

### Glucose uptake

HepG2 cells were seeded in 24-well plates for 24 hr and then incubated with DMEM containing 5.5 or 33 mM glucose, 0 or 10^−7^ M insulin, 1% FBS and PSK (1000 μg/ml) or TV LH-1 ePSP (0, 100, 500, or 1000 μg/ml). After incubation for 24 hr, the medium was removed and replaced by DMEM containing 5.5 mM or 33 mM glucose, 1% FBS, 0 (NG model and HG model) or 10^−7^ M insulin (NG model+Ins1, HG model+Ins1 and HGI model) and 100 μM of 2-NBDG for 1 hr. The 1 hr insulin stimulation was to test the insulin response of these models. Due to the HGI model that needs insulin to maintain its characteristics, we continuously treated cells with insulin during the experimental period. The use of 2-NBDG for glucose uptake was based on the studies of Zou et al. [17] and O’Neil et al. [18].

After incubation with 2-NBDG for 1 hr, glucose uptake reaction was stopped by removing medium and washing with phosphate-buffered saline (PBS). Subsequently, cells were resuspended by trypsin-EDTA, neutralized by DMEM containing 10% FBS, treated with propidium iodide (PI, 5 μg/ml), and measured by flow cytometer (Cytomics FC500, Beckman Coulter, Inc., Indianapolis, IN). For the determination of glucose uptake, a minimum of 10^4^ cells per sample was collected and analyzed to estimate the percentage of HepG2 cells that absorbed 2-NBDG, i.e., an index of glucose uptake. Using flow cytometer, 10 000 cells were collected and the percentages of glucose uptake in HepG2 cells without any treatment were adjusted to approximately 50% of live cells that absorbed 2-NBDG without PI for the NG, HG and HGI models, individually, as the baselines. For each model, the individual voltage setting of flow cytometer was used to assess the effects of insulin, PSK or ePSP on glucose uptake.

### Glycogen contents

HepG2 cells were seeded in 6-well plates for 24 hr and then incubated with DMEM containing 5.5 or 33 mM glucose, 0 or 10^−7^ M insulin, 1% FBS, and PSK (1000 μg/ml) or TV LH-1 ePSP (0, 100, 500, or 1000 μg/ml). After incubation for 24 hr, cells were washed with PBS, resuspended by trypsin-EDTA, neutralized by DMEM containing 10% FBS, lyzed in 30% KOH at 100°C for 30 minutes and extracted by 99.9% ethanol at 4°C for 30 minutes. The precipitates were added with an equal volume of ddH_2_O and 5% phenol solution immediately followed by the addition of H_2_SO_4_ [23]. After hydrolysis, glycogen content was determined by the absorbance at 490 nm.

### Western blot analysis for proteins in glucose metabolism and insulin signaling pathway

HepG2 cells were seeded in 100-mm dishes for 24 hr and then incubated with DMEM containing 5.5 or 33 mM glucose, 0 or 10^−7^ M insulin, 1% FBS, and PSK (1000 μg/ml) or LH-1 ePSP (0, 100, 500, or 1000 μg/ml). After incubation for 24 hr, cells were treated with media with or without 10^−7^ M insulin for 1 hr, collected, and homogenized in 50 ml of lysis buffer A (10 mM Tris/HCl pH7.0, 150 mM NaCl, 2 mM EDTA, 1 mM Na_3_VO_4_, 0.2 mM PMSF, 50 μM NaF, 10 μg/ml aprotinin, 10 μg/ml leupeptin, 10μg/ml pepstatin, and 1% Triton X-100) and incubated at 4°C for 30 minutes. Cell lysates were separated by centrifugation at 16000 ×g at 4°C for 20 minutes. The supernatant was collected as cytosol protein, and the pellet was resuspended in 50 ml of lysis buffer B (10 mM Tris/HCl pH7.0,150 mM NaCl, 1 mM EDTA, 1 mM EGTA, 0.5 mM Na_3_VO_4_,0.1 mM PMSF, 25 μM NaF, 10 μg/ml aprotinin, 10 μg/ml leupeptin, 10 μg/ml pepstatin, 1%Triton X-100, and 0.5% NP40), incubated at 4°C for 30 minutes, and centrifuged at 16000 ×g at 4°C for 20 minutes. The supernatant was collected as membrane protein.

Protein concentrations of the extracts were determined using the BCA protein-assay kit (Pierce, Rockford, IK, USA) with bovine serum albumin as the standard. Thirty micrograms of proteins were separated by 7.5 or 10% of sodium dodecyl sulfate-polyacrylamide gel electrophoresis (SDS-PAGE) and transferred onto a polyvinylidene difluoride (PVDF) membrane (PerkinElmer, Zaventem, Belgium). The PVDF membrane was blocked by incubation in Tris-buffered saline Tween-20 (TBST) solution containing 5% skim milk at room temperature for 1 hr, followed by an overnight incubation at 4°C with polyclonal anti-human/rat antibodies of glucokinase, glycogen synthase kinase (GSK) 3β, phosphoenolpyruvate carboxykinase (PEPCK), glucose 6-phosphatase (G6Pase), phosphorylated insulin receptor (p-IR) β, total IRβ, insulin receptor substrate (IRS) 1 and 2, phosphorylated phosphatidylinositol-3-kinases (p-PI3K), phosphorylated Akt (p-Akt), total Akt, glucose transporter (Glut) 1 and 2, phosphorylated AMP-activated protein kinase (p-AMPK), and total AMPK (Cell Signaling Technology, Danvers, MA, USA) or monoclonal anti-human/rat antibodies of β-actin (Millipore, MA, USA) and glucokinase (Santa Cruz, CA, USA) at a dilution of 1:1000 to 1:50 000.

After being washed 3 times by TBST solution, the membranes were incubated with horseradish peroxidase-labeled goat anti-mouse IgG or goat anti-rabbit IgG antibodies at the dilution of 1:5000 to 1:25 000 for 1 hr at room temperature. Signals were visualized by incubating in chemiluminescent horseradish peroxidase substrate (ECL, Millipore, MA, USA) and by exposing to X-ray film. Band densities were determined using the UVP BioSpectrum® CCD Imaging System (Upland, CA, USA).

### Statistical analysis

Data are expressed as mean ± standard error of the means (SEM). The normality of distribution and homogeneity of variance of the data were tested by Levene's test using SPSS. Log transformation was used to correct-normalize the skewed data from the western blot analysis. The differences among groups were compared by one-way analysis of variance (ANOVA) using the SAS general linear models program. Group means were considered to be significantly different at P < 0.05, as determined by protective least significant difference (LSD) when the ANOVA indicated an overall significant treatment effect of P < 0.05.

## Results

### HepG2 cells of the HG and HGI models had lower glucose uptake and insulin sensitivity

**Fig 1:**
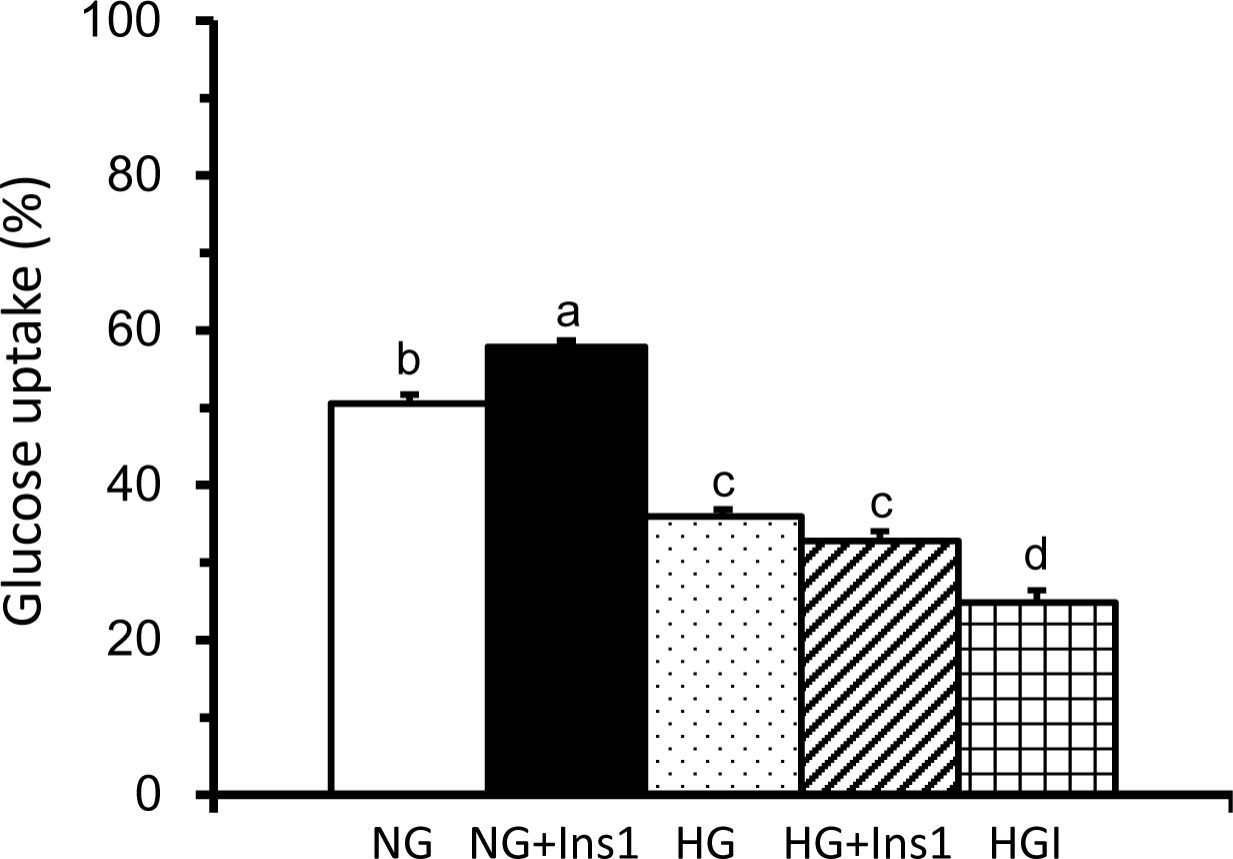
Glucose uptake of HepG2 cells in the NG, HG and HGI models with or without insulin stimulation. Cells were incubated in DMEM with 5.5 mM glucose, 33 mM glucose, or 33 mM glucose plus 10^−7^ M insulin for 24 hr followed by 0 (NG model and HG model) or 10^−7^ M insulin stimulation (NG model+Ins1, HG model+Ins1 and HGI model) for 1 hr. Glucose uptake was determined by flow cytometer as the percentages of HepG2 cells absorbed with fluorescently-labeled 2-NBDG without propidium iodide (PI). The percentages of glucose uptake in the NG model without any stimulation was adjusted to approximately 50% of cells absorbed 2-NBDG without PI. To compare the glucose uptake of different models with the NG model, the voltage setting of flow cytometer was consistent. Values are means ± SEM, n = 9 for each group. Values with completely different lowercase superscript letters indicate significant differences among the models (one-way ANOVA with LSD, P < .05).

The results of glucose uptake of the NG, HG and HGI models are shown in Fig 1. As shown in Fig 1, the cellular uptake of 2-NBDG in the NG model without insulin stimulation was adjusted to approximately 50%. The HG model had significantly decreased glucose uptake compared to the NG model, and the HGI model had further decreased glucose uptake compared to the HG model. In the NG model, insulin stimulation significantly increased glucose uptake, i.e., the NG+Ins1. The insulin stimulated glucose uptake was undetected in the HG and HGI models. These results suggest that HepG2 cells of the HG and HGI models had decreased glucose uptake and insulin sensitivity.

### TV LH-1 ePSP did not affect HepG2 cell viability in the concentration less than 2000 μg/ml

Cell viability of HepG2 cells was decreased by 20% and 50% with 2000 and 4000 μg/ml of PSK, but not affected by 0.01 to 1000 μg/ml of PSK after incubation for 24, 48, and 72 h compared to cells without PSK (S1A Fig). In addition, cell viability of HepG2 cells was decreased by 30% after 4000 μg/ml of TV LH-1 ePSP, but not affected by TV LH-1 in the concentrations less than 2000 μg/ml after incubation for 24, 48, and 72 h (S1B Fig).

### TV LH-1 ePSP increased glucose uptake and glycogen content of HepG2 cells in the HG and HGI models

**Fig 2:**
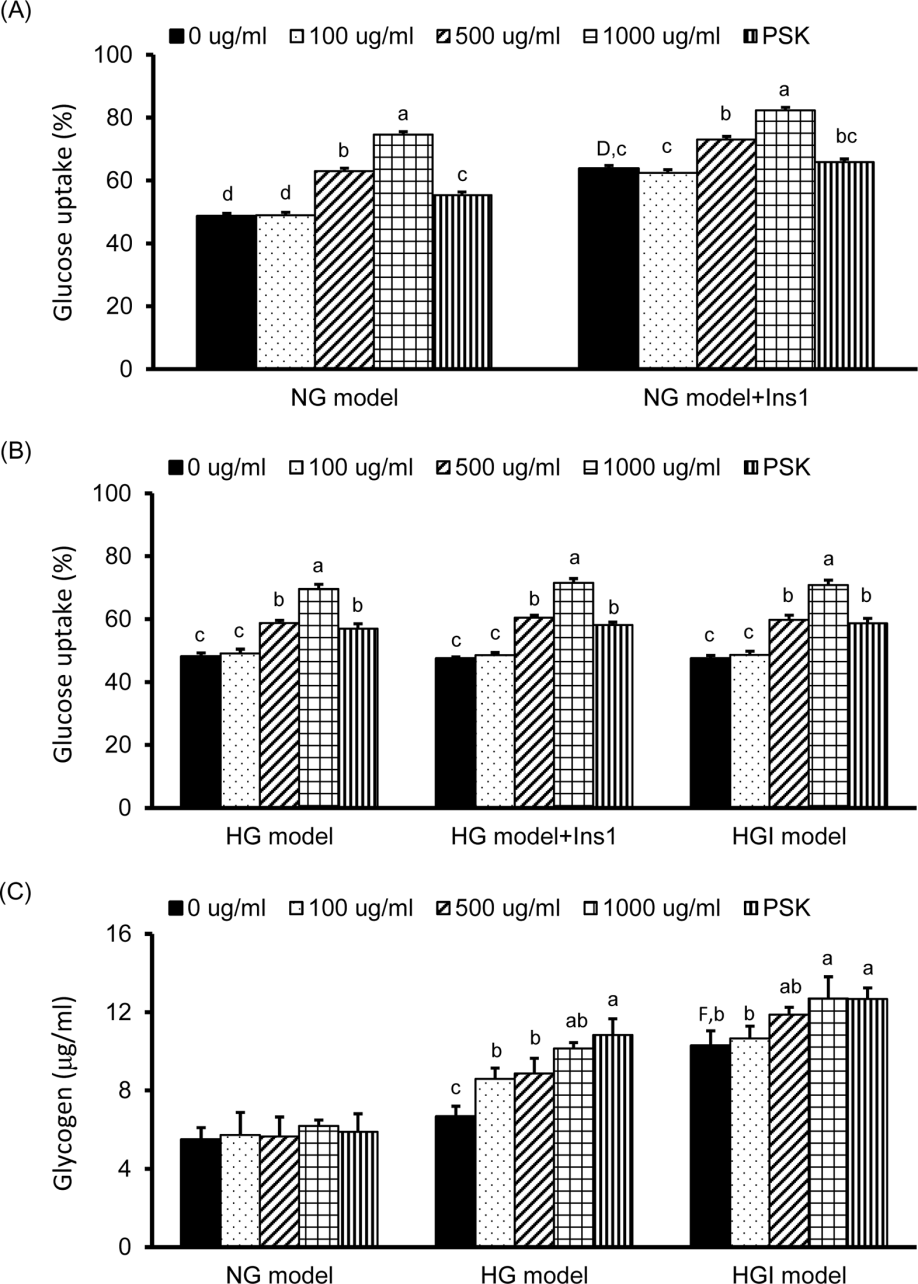
Glucose uptake and glycogen storage of HepG2 cells with TV LH-1 ePSP, PSK or insulin treatment. Glucose uptake (A and B) and glycogen storage (C) of HepG2 cells incubated in DMEM with 5.5 mM glucose (NG model), 33 mM glucose (HG model), or 33 mM glucose plus 10^−7^ M insulin (HGI model) and treated with TV LH-1 ePSP (0, 100, 500, or 1000 μg/ml) or PSK (1000 μg/ml) for 24 hr followed by 0 or 10^−7^ M insulin treatment for 1 hr. Glucose uptake was determined by flow cytometer as the percentages of HepG2 cells absorbed with fluorescently-labeled 2-NBDG without propidium iodide (PI) as described in the Methods and Materials section. Values are means ± SEM, n = 12 for each group. Values with uppercase superscript letters D and F indicated that the NG model+ins1 and HGI model with no treatment (i.e., 0 μg/ml) were significantly different from the NG model (0 μg/ml); and those with different lowercase superscript letters indicate significant differences within each model (one-way ANOVA with LSD, P < .05).

The effects of TV LH-1 ePSP and PSK on glucose uptake in the NG, HG, and HGI models are shown in Fig 2. In the NG model without insulin stimulation (the left-side bars in Fig 2A), glucose uptake of HepG2 cells was increased 30% to 50% by 500 and 1000 μg/ml of TV LH-1 ePSP and approximately 12% by 1000 μg/ml of PSK compared to that of cells without any treatment. In the NG model with 1 hr insulin stimulation, i.e., the NG+Ins1 (the right-side bars in Fig 2A), glucose uptake was increased approximately 30% compared to the NG model without any treatment and was increased 15 to 30% by 500 and 1000 μg/ml of TV LH-1 ePSP. However, PSK did not significantly alter glucose uptake in the NG model with 1 hr insulin stimulation.

In the HG model, glucose uptake was significantly increased approximately 20 to 50% by 24 hr of 500 and 1000 μg/ml of TV LH-1 ePSP and approximately 20% by 24 hr of 1000 μg/ml of PSK with or without 1 hr insulin stimulation compared to that of cells without any treatment (Fig 2B). In the HGI model, glucose uptake was significantly increased approximately 20 to 45% by 24 hr of 500 and 1000 μg/ml of TV LH-1 ePSP and approximately 20% by 1000 μg/ml of PSK treatment (Fig 2B). However, glucose uptake was not significantly altered in the HG model with insulin stimulation, i.e., the HG+Ins1, and the HGI model. These results suggested that the HepG2 cells in the HG and HGI models were insulin resistant. In addition, TV LH-1 ePSP had the abilities to increase glucose uptake in a dose-dependent response in all of the 3 models.

HepG2 cells in the HGI model, not in the HG model, had a significantly increased amount of glycogen compared to those in the NG model. In addition, the amount of glycogen in the cells was significantly increased by 24 hr of TV LH-1 ePSP and PSK treatments in the HG and HGI models, not in the NG model (Fig 2C).

### TV LH-1 ePSP increased IRS2 protein expression in the HG and HGI models

**Fig 3:**
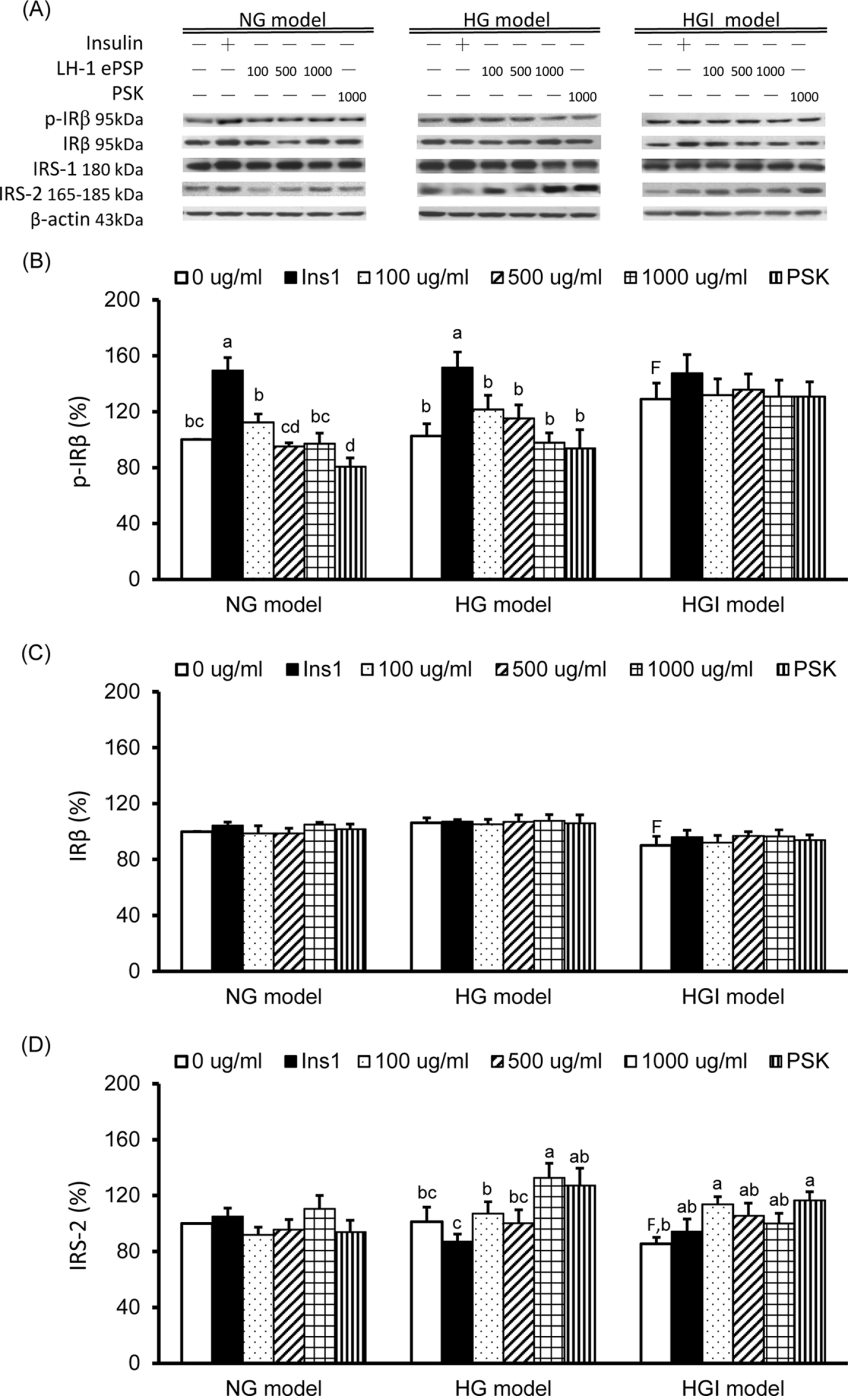
Protein expression of insulin receptor and insulin receptor substrates. Western blot analysis was used to detect phosphorylated insulin receptor (p-IR) b, total IRb, insulin receptor substrate (IRS) 1 and 2, and the internal control β-actin (A). Protein expression of p-IRb (B), total IRb (C) and IRS2 (D). Protein quantification was carried out by densitometric analysis, normalized by the internal control β-actin, and calculated as the percentages of the NG model without any treatment (0 μg/ml). Values are means ± SEM, n = 9 for each group. Values with uppercase superscript letter F indicated that the HGI model with no treatment (i.e., 0 μg/ml) was significantly different from the NG model (0 μg/ml); and those with different lowercase superscript letters indicate significant differences within each model (one-way ANOVA with LSD, P < .05).

The results of western blot analysis in p-IRβ (the active form), total IRβ, IRS1, and IRS2 are shown in Fig 3. In cells with no treatment, the protein expression of p-IRβ (Fig 3B) was significantly higher and that of total IRβ (Fig 3C) and IRS2 (Fig 3D) were significantly lower in the HGI model than in the NG model. After stimulation with insulin for 1 hr, HepG2 cells had significantly increased p-IRβ expression in both the NG and HG models, but not in the HGI model. TV LH-1 ePSP did not have a significant impact on the expression of p-IRβ and total IRβ in all the models. However, 1000 μg/ml of PSK significantly decreased p-IRβ expression in the NG model. The expression of IRS2 was significantly increased by 1000 μg/ml of TV LH-1 ePSP in the HG model and by 100 μg/ml of TV LH-1 ePSP and 1000 μg/ml of PSK in the HGI model. The expression of IRS1 was not significantly different among 3 models and was not significantly altered by 1 hr insulin stimulation, TV LH-1 ePSP, or PSK.

**Fig 4:**
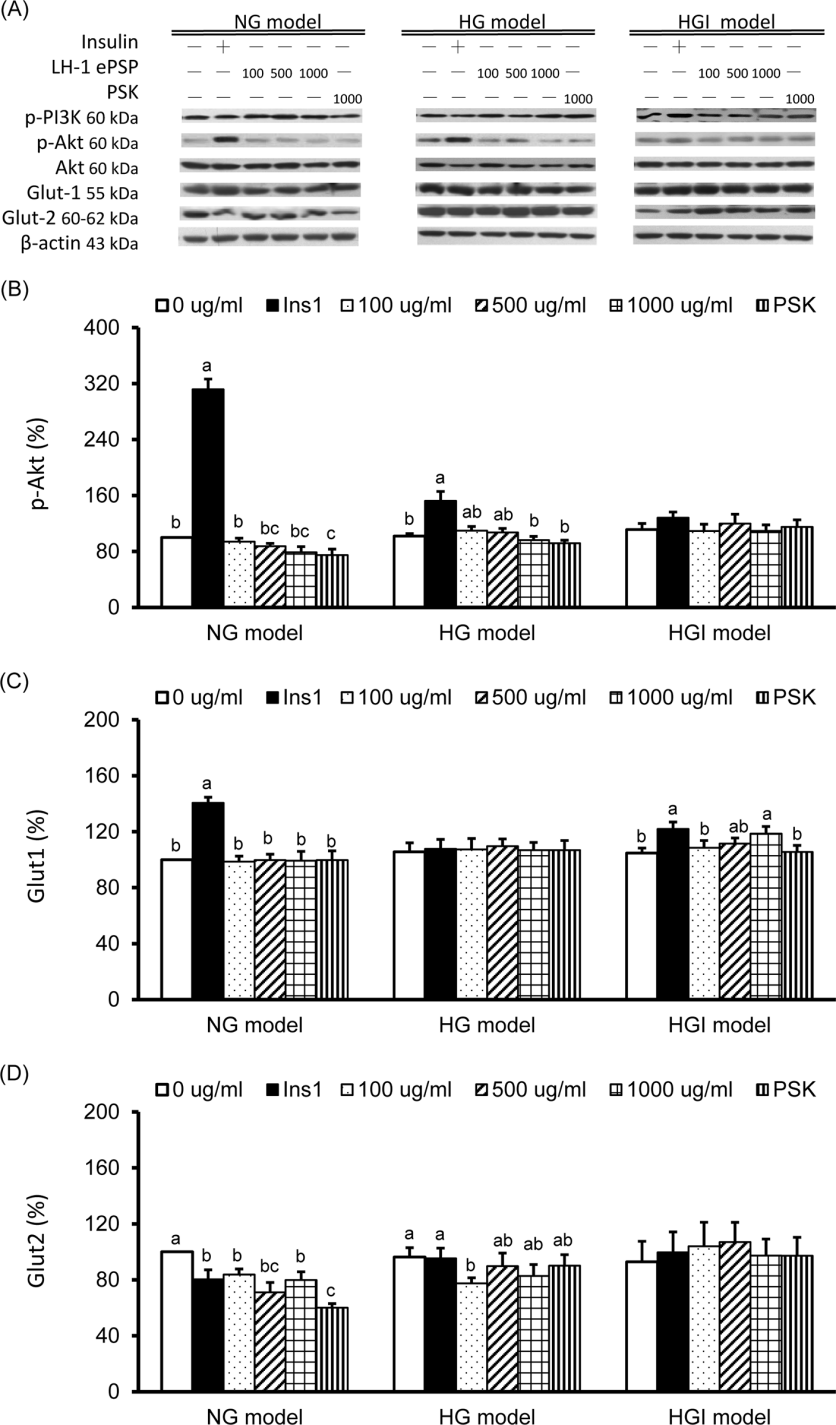
Protein expression of molecules in the insulin signaling pathway and glucose transporters. Western blot analysis was used to detect phosphorylated phosphatidylinositol-3-kinases (p-PI3K), phosphorylated Akt (p-Akt), total Akt, glucose transporter (Glut) 1, Glut2 and the internal control β-actin (A). Protein expression of p-Akt (B), Glut1 (C) and IRS2 (D). Protein quantification was carried out by densitometric analysis, normalized by the internal control β-actin, and calculated as the percentages of the NG model without any treatment (0 μg/ml). Values are means ± SEM, n = 9 for each group. Values with different lowercase superscript letters indicate significant differences within each model (one-way ANOVA with LSD, P < .05).

The protein expression of molecules in the insulin signaling pathway are shown in Fig 4. The expression of p-PI3K and total Akt were not significantly different among 3 models and were not significantly altered by 1 hr insulin stimulation, TV LH-1 ePSP or PSK. The p-Akt (the active form) protein expression was significantly increased by 1 hr insulin treatment in the NG and HG models, not the HGI model, and was significantly decreased by 1000 μg/ml of PSK in the NG model (Fig 4B). The Glut1 expression of the cell membrane was significantly increased by 1 hr insulin treatment in the NG and HGI models and by 1000 μg/ml of TV LH-1 ePSP in the HGI model (Fig 4C). The Glut2 expression of the cell membrane was significantly decreased by 1 hr insulin stimulation, TV LH-1 ePSP and PSK in the NG model and by 100 μg/ml of TV LH-1 ePSP in the HG model (Fig 4D).

### TV LH-1 ePSP increased glucokinase protein expression in the HG model and decreased GSK3β and increase G6Pase protein expression in the HGI model

**Fig 5:**
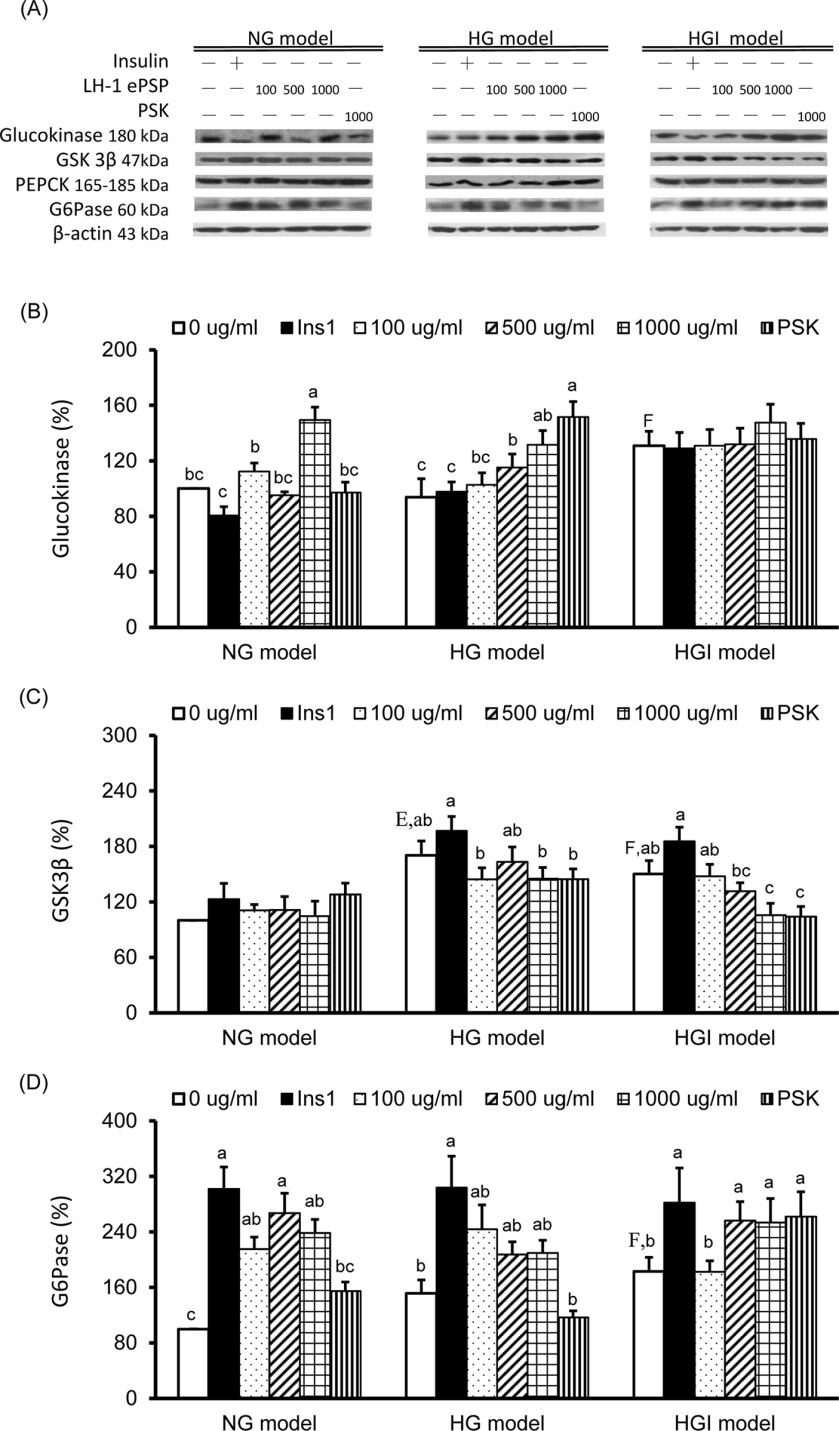
Protein expression of molecules in glucose homeostasis-associated enzymes. Western blot analysis was used to detect glucokinase, glycogen synthase kinase (GSK) 3b, phosphoenolpyruvate carboxykinase (PEPCK), glucose 6-phosphatase (G6Pase) and the internal control β-actin (A). Protein expression of glucokinase (B), GSK3b (C) and G6Pase (D). Protein quantification was carried out by densitometric analysis, normalized by the internal control β-actin, and calculated as the percentages of the NG model without any treatment (0 μg/ml). Values are means ± SEM, n = 9 for each group. Values with uppercase superscript letters E and F indicated that the NG model+ins1 and HGI model with no treatment (i.e., 0 μg/ml) were significantly different from the NG model (0 μg/ml); and those with different lowercase superscript letters indicate significant differences within each model (one-way ANOVA with LSD, P < .05).

To evaluate the effects of TV LH-1 ePSP on glucose homeostasis, such as glycolysis, glycogen synthesis, and gluconeogenesis, we measured the protein expressions of glucokinase, GSK3β, PEPCK, and G6Pase (Fig 5A). The glucokinase expression was significantly higher in the HGI model than in the NG model and was significantly increased by 1000 μg/ml of TV LH-1 ePSP in the NG model and by 500 and 1000 μg/ml of TV LH-1 ePSP and 1000 μg/ml of PSK in the HG model (Fig 5B). The expression of GSK3β was significantly increased in the HG and HGI models compared to the NG model (Fig 5C). The expression of PEPCK was not significantly different among 3 models and was not significantly altered by 1 hr insulin stimulation, TV LH-1 ePSP, or PSK. The G6Pase expression was significantly higher in the HGI model than in the NG model and was significantly increased by 1 hr insulin treatment in all 3 models, by 100, 500, and 1000 μg/ml of TV LH-1 ePSP in the NG model and 500 and 1000 of μg/ml of TV LH-1 ePSP and 1000 μg/ml of PSK in the HGI model (Fig 5D).

### TV LH-1 ePSP increased AMPK activation in HepG2 cells of the HG and HGI models

**Fig 6:**
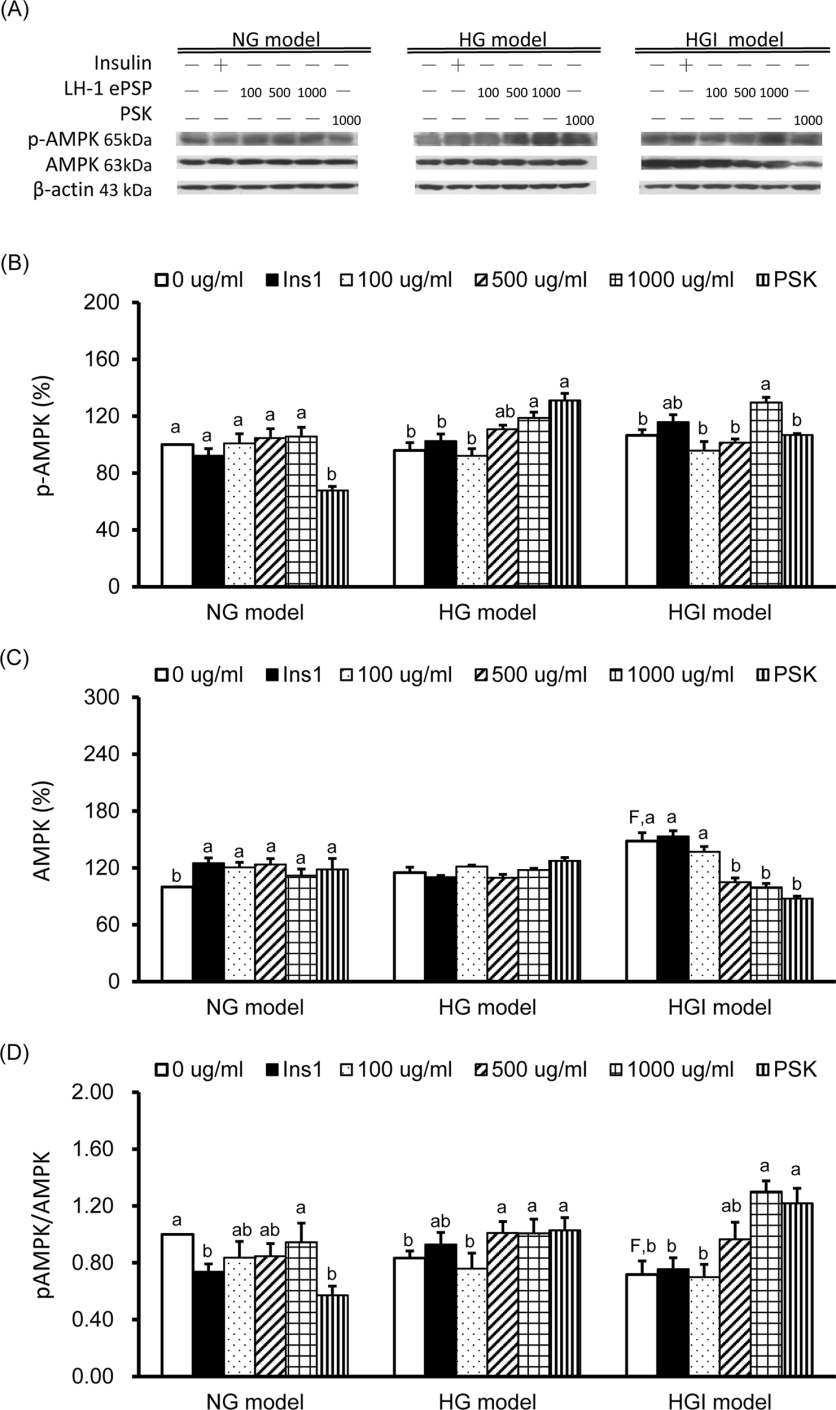
Protein expression of molecules in energy homeostasis. Western blot analysis was used to detect phosphorylated AMP-activated protein kinase (p-AMPK), total AMPK, and the internal control β-actin (A). Protein expression of p-AMPK (B), total AMPK (C), and AMPK activation, i.e., the ratio of p-AMPK and total AMPK (D). Protein quantification was carried out by densitometric analysis, normalized by the internal control β-actin, and calculated as the percentages of the NG model without any treatment (0 μg/ml). Values are means ± SEM, n = 9 for each group. Values with uppercase superscript F indicated that the HGI model with no treatment (i.e., 0 μg/ml) were significantly different from the NG model (0 μg/ml); and those with different lowercase superscript letters indicate significant differences within each model (one-way ANOVA with LSD, P < .05).

To evaluate the effects of TV LH-1 ePSP on energy homeostasis, we measured the protein expression of p-AMPK (the active form) and total AMPK and the ratio of p-AMPK to total AMPK (Fig 6). The results showed that p-AMPK protein expression was significantly decreased by 1000 μg/ml of PSK in the NG model, increased by 1000 μg/ml of TV LH-1 ePSP and PSK in the HG model, and increased by 1000 μg/ml of TV LH-1 ePSP in the HGI model (Fig 6B). Total AMPK was significantly increased in the HGI model compared to the NG model, increased by 1 hr insulin stimulation, TV LH-1 ePSP, and PSK in the NG model and decreased by 500 and 1000 μg/ml of TV LH-1 ePSP and 1000 μg/ml of PSK in the HGI model (Fig 6C). The activation of AMPK was significantly decreased in the HGI model compared to the NG model, decreased by 1 hr insulin stimulation and 1000 μg/ml of PSK in the NG model, increased by 500 and 1000 μg/ml of TV LH-1 ePSP and 1000 μg/ml of PSK in the HG model, and increased by 1000 μg/ml of TV LH-1 ePSP and PSK in the HGI model (Fig 6D).

## Discussion

DM is a metabolic disorder characterized by hyperglycemia, and its persistently increased prevalence results in rapidly rising global medical costs [1]. Various alternative approaches, including the use of functional foods and nutraceuticals, have been developed to prevent and/or control the diabetes-associated complications. Using HepG2 cells, high glucose, high insulin, and a fluorescent-labeled glucose analog, 2-NBDG, we have developed a non-isotopic, relatively safe and sensitive *in vitro* tool to rapidly screen substances for anti-hyperglycemic or anti-diabetic activity. In addition, we have demonstrated that TV LH-1 ePSP may have the ability to regulate glucose homeostasis by significantly increasing glucose uptake in a dose-dependent manner (up to 1000 μg/ml) in insulin resistant HepG2 cells via the activation of AMPK and glycogen synthesis in an insulin-independent manner.

The liver plays a crucial role in glucose homeostasis, which involves the regulation of glycolysis, gluconeogenesis, glycogenesis, and glycogenolysis in responding to a variety of physiological conditions, ranging from hypoglycemia to hyperglycemia, controlled by insulin, glucagon, and other hormones [24]. To investigate the glucose uptake of HepG2 cells, we used 2-NBDG, a fluorescent derivative of glucose, as the indicator. This indicator has been shown to be incorporated into living cells via the glucose transporting system, is phosphorylated by hexokinase, and enters the glycolytic pathway [25]. Our preliminary study showed that 100 mM of 2-NBDG within 180 minutes may reach a dynamic equilibrium level of entry and decomposition of 2-NBDG in HepG2 cells, and 60 minutes of incubation may reach approximately 50% uptake of 2-NBDG (data not shown) as in agreement with the results shown by Zou et al. [17]. We subsequently incubated HepG2 cells with high glucose and high glucose plus high insulin levels to mimic the physiological conditions of the liver in patients with type 1 and type 2 DM, respectively, as reported in the study of Nakajima et al [21].

We confirmed that insulin may significantly increase glucose uptake in the NG model, i.e., HepG2 cells incubated with 5.5 mM (100 mg/ml) of glucose (Figs 1 and 2A). In the cells exposed to higher glucose levels (the HG and HGI models) the efficacy of insulin-stimulated glucose uptake was impaired (Figs 1 and 2B). Concurrently, molecules in the insulin signaling pathway were also affected in the HG and HGI models where insulin stimulation increased. For example, insulin stimulation increased p-IRβ protein expression (Fig 3A) in the HG but also NG models. However, the effects of insulin on the downstream protein pAkt (Fig 4A) and membrane Glut1 were decreased and negated in the HG model when compared to the NG model (Fig 4B). In addition, insulin stimulation did not alter p-IRβ nor p-Akt protein expression in the HGI model, suggesting impairment of the insulin signaling pathway.

The anti-hyperglycemic or anti-diabetic activity of mushrooms has been demonstrated in *in vitro* and *in vivo* studies, which is closely related to their copious content of dietary fibre, polysaccharides, and secondary metabolites [4]. The polysaccharide constituent of *Trametes versicolor* has been used as a concurrent adjuvant for cancer therapy and has been found to possess various biological activities. Our previous study demonstrated that TV LH-1 ePSP may have protective effects on bone micro-architecture and quality in a diabetes-induced bone-loss model [14], but the effect of TV on hyperglycemia is yet to be fully elucidated. In the present study, TV LH-1 ePSP may increase glucose uptake in HepG2 cells, with or without insulin stimulation, in a dose-dependent manner (Fig 2), suggesting an insulin-independent activity in increasing glucose uptake. We also found that TV LH-1 ePSP had greater activity in elevating glucose uptake than PSK. Considering the productivity, the submerged fermentation produces more extracellular than intracellular polysaccharopeptides. These results suggest that TV LH-1 ePSP is a superior candidate for industry to develop a functional food with anti-hyperglycemic or anti-diabetic activity.

To investigate the mechanism of LH-1 ePSP in increasing glucose uptake, we analyzed certain molecules involved in the insulin signaling pathway, i.e., IRβ, IRS 1 and 2, PI3K, and Akt. As reported in previous studies, HepG2 cells incubated in 30 mM of glucose and 100 nM of insulin had diminished levels of p-Akt [26]. Our results showed that insulin significantly increased the protein expression of p-IRβ (Fig 3B) and the downstream signaling molecule p-Akt (Fig 4B) in HepG2 cells with normal and high glucose concentrations. However, TV LH-1 ePSP and PSK did not significantly activate IRβ, IRS-1, p-Akt, or p-PI3K (Fig 4A). These results implied that the effect of TV LH-1 ePSP and PSK on elevating glucose uptake may not be mediated by the insulin signaling pathway.

Glucose uptake of mammalian cells is catalyzed by a family of glucose transport proteins. For example, Glut1 is expressed in most cells at low levels and responsible for low basal glucose uptake and Glut2 is highly expressed in the liver in an insulin-independent system and has high capacity, but low affinity, for glucose [27]. In the present study, we confirmed that insulin significantly increased membrane Glut1, not Glut2 expression in HepG2 cells of the NG model (Fig 4C). In addition, we found that TV LH-1 ePSP increased membrane Glut1 in the HGI model and decreased membrane Glut2 in the NG and HG models. These results indicated that the TV LH-1 ePSP-elevated glucose uptake in HepG2 cells may be not mainly due to the changes in glucose transporters.

Defective insulin signaling and development of insulin resistance in the liver may affect energy balance and metabolism [28]. Therefore, we further investigated the alterations of the key enzymes in glucose metabolism including glycolysis, gluconeogenesis, glycogenesis, and glycogenolysis. In the present study, compared to the NG model, the HGI model had increased glucokinase (Fig 5A), GSK3β, the enzymes control glycolysis and glycogen synthesis via the inhibition of IRS-1 and Akt activation, and G6Pase (Fig 5C), the enzyme completes the final step in gluconeogenesis and glycogenolysis. The glycogen storage, determined after 24 hr of incubation, was significantly increased in the HGI model compared to the NG model, suggests increased glycogen synthesis. We speculate that there was an imbalance in the energy homeostasis in the HGI model, as shown in the decreased AMPK activation, which resulted in the increased glycogen accumulation in the condition with decreased glucose uptake. The increased glycogen synthesis in the HGI model may be similar to the hepatic glycogenosis occurring in patients with type 2 diabetes mellitus who had concomitant presence of insulin and excess glucose [29]. When treated with TV LH-1 ePSP, HepG2 cells in the HGI model had decreased GSK3β, the inhibitor of glycogen synthase, and increased G6Pases suggesting a further increased glycogen synthesis. TV LH-1 ePSP also increased glucokinase in the NG and HG models. All these results implied that TV LH-1 ePSP-elevated glucose uptake may be used for glycogen synthesis.

In addition to glycogen synthesis, HepG2 cells use glucose for energy production. To investigate glucose utilization, we further investigated the activation of AMPK, the key player in maintaining cellular energy homeostasis via the stimulation of glucose uptake, glycolysis and fatty acid oxidation and inhibition of gluconeogenesis and lipid synthesis [19]. The HGI model had an increased total AMPK protein (Fig 6B) and a decreased ratio of phosphorylated to total AMPK (Fig 6C), suggesting an inefficient energy metabolism. When treated with TV LH-1 ePSP and PSK, HepG2 cells in the HG and HGI models had increased AMPK activation. The increased glycogen synthesis and AMPK activation revealed that TV LH-1 ePSP and PSK may increase the utilization of glucose for energy production.

Despite the encouraging results, there are several limitations in the present study. First, the liver is formed by different types of cells, and the HepG2 cell line can only represent parenchymal cells. In addition, HepG2 cells have lower metabolic capacities compared with primary cells and have been found to express less drug-metabolizing enzymes [30]. However, the HepG2 cell line is a useful and reliable tool with abundance of well-known characteristics which provides a reproducible human system, such as synthesis and secretion of the major plasma proteins. Secondly, the NG, HG, and HGI models we established can only partially represent the increased glucose and/or insulin levels of the diabetic patients, which may not completely mimic the complicated biological conditions, such as the low and chronic inflammation and dysregulated immune response. However, this *in vitro* platform may be used to screen for the anti-hyperglycemic candidates without using animals to test the bioactivities of the unknown materials, but in addition, a known drug having anti-hyperglycemic activity may be used as a positive control. Thirdly, orally administered TV LH-1 ePSP may be digested by intestinal enzymes or may affect the profile of gut microbiota in animals and humans [31]. Animal and clinical studies are still needed to confirm the anti-hyperglycemic activity of TV LH-1 ePSP.

## Conclusions

The present study has established an *in vitro* HepG2 cell platform with insulin resistance. Using flow cytometry to measure the percentages of fluorescent 2-NBDG absorbed by HepG2 cells, this *in vitro* platform can provide a rapid and accurate tool to screen the effects and mechanism of potential products on regulating glucose homeostasis before further costly investigation is initiated. In this study the HG and HGI models had decreased glucose uptake, impaired insulin signaling pathway, as shown in the elevated activation of IRβ expression and decreased IRS-2 and phosphorylated Akt, and unchanged or even decreased activation of AMPK, as shown in the ratio of phosphorylated to total AMPK. These results suggest that the HG and HGI models presented a condition similar to insulin resistance. In addition, we have demonstrated that TV LH-1 ePSP may elevate glucose uptake and regulate glucose homeostasis in hepatocytes with insulin resistance via the activation of AMPK and glycogen synthesis in an insulin-independent manner, as shown in the decreased GSK3, increased G6Pase and the ratio of phosphorylated to total AMPK and unchanged phosphorylated IR and Akt. These results suggest that TV LH-1 ePSP has a great potential to be an anti-hyperglycemic functional food.

## Supporting information

S1 Fig Cell viability of HepG2 cells with PSK or TV LH-1 ePSP. Cell viability of HepG2 cells incubated in DMEM with 5.5 mM glucose, 10% FBS and 0, 0.01, 0.1, 1, 10, 100, 500, 1000, 2000, or 4000 μg/ml of PSK (A) or TV LH-1 ePSP (B) for 24, 48 and 72 hr. Values are means ± SEM, n = 10–12 for each treatment.
(TIF)
