# Supplementary figures and images for: Potential activities and mechanisms of extracellular polysaccharopeptides from fermented Trametes versicolor on regulating glucose homeostasis in insulin-resistant HepG2 cells
Source: PLoS One. 2018 Jul 19;13(7):e0201131. doi: 10.1371/journal.pone.0201131 (PMC6053205; doi:10.1371/journal.pone.0201131)

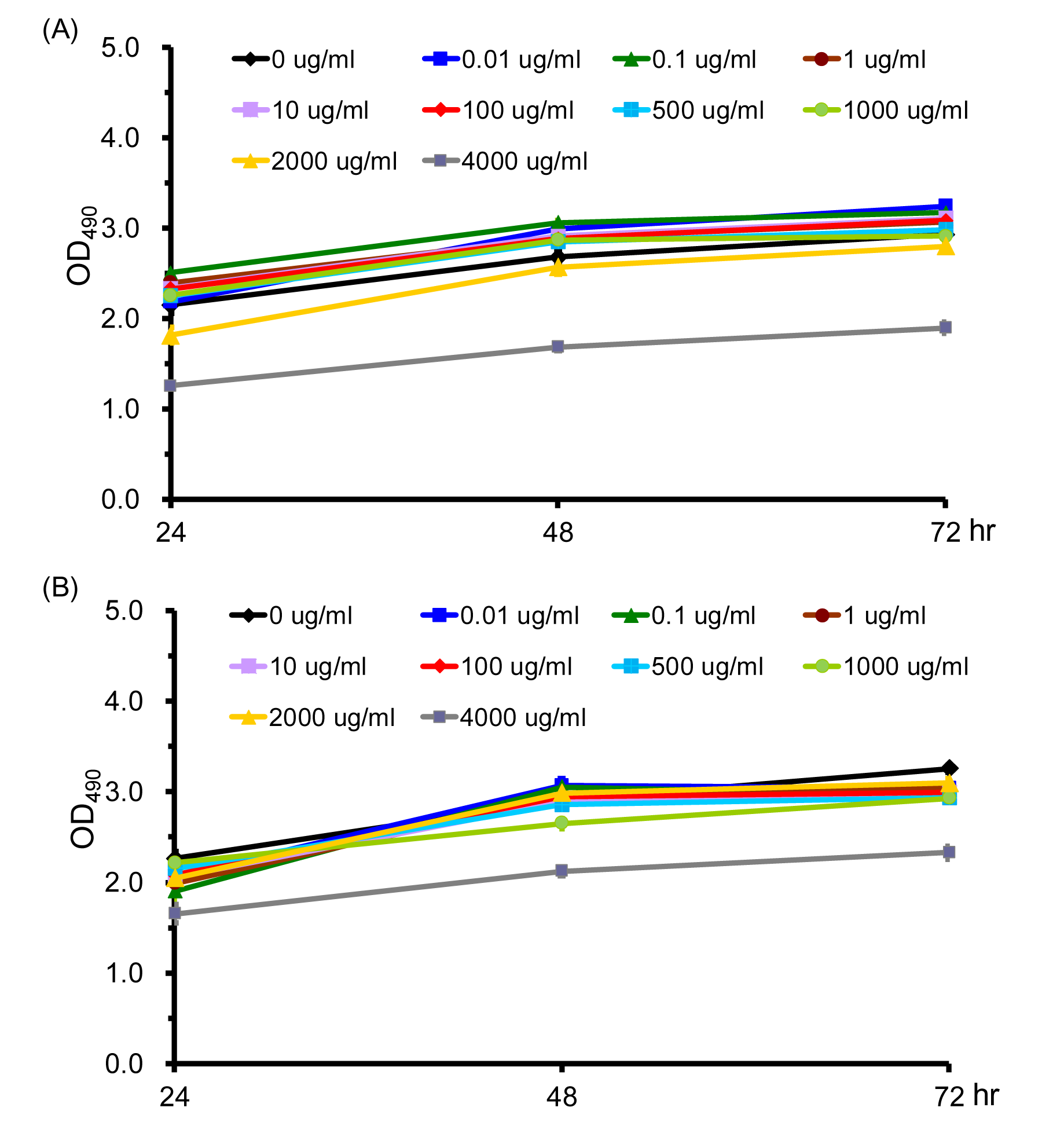

Supplement: S1 Fig — Cell viability of HepG2 cells incubated in DMEM with 5.5 mM glucose, 10% FBS and 0, 0.01, 0.1, 1, 10, 100, 500, 1000, 2000, or 4000 μg/ml of PSK (A) or TV LH-1 ePSP (B) for 24, 48 and 72 hr. Values are means ± SEM, n = 10–12 for each treatment. (TIF) [file pone.0201131.s001.tif]
